# A Review of Research on Throwing Biomechanics, Upper Extremity Injuries, and Treatment of Throwing Injuries in Professional Baseball and Football

**DOI:** 10.1177/23259671251407244

**Published:** 2026-01-27

**Authors:** Kushagra Tewari, Benjamin Sun, Mathangi Sridharan, Thomas Olson, Stone Streeter, Paul Walker, Sharon Hame, Frank Petrigliano

**Affiliations:** * Department of Orthopaedic Surgery, David Geffen School of Medicine at University of California, Los Angeles, Los Angeles, California, USA; † Wayne State School of Medicine, Detroit, Michigan, USA; § Weill Cornell Medicine, New York, New York, USA; Investigation performed at the University of California, Los Angeles (UCLA), Los Angeles, California, USA

**Keywords:** baseball injuries, football injuries, biomechanics, UCL injury, AC joint injury, SLAP tear

## Abstract

**Background:**

Throwing is a fundamental skill in many sports that requires intricate biomechanical coordination to achieve maximum performance and minimize injury. Baseball pitchers and football quarterbacks have distinct throwing motions that involve unique demands on the upper extremity, which ultimately contribute to injury, especially in cases of overuse.

**Purpose:**

To compare the throwing biomechanics, incidence, and types of injuries sustained and their respective treatments between baseball pitchers and football quarterbacks.

**Study Design:**

Narrative review.

**Methods:**

Comprehensive literature review examining biomechanical differences between baseball pitching and football quarterback throwing motions, analyzing injury patterns and prevalence in both sports, and evaluating current treatment approaches for throwing-related upper extremity injuries. Multiple databases (Google Scholar and PubMed) were queried for peer-reviewed publications related to baseball/football throwing biomechanics (biomechanics, kinematics, kinetics, electromyography, throwing phases, arm slot, external rotation, varus torque, kinetic chain), injury entities (ulnar collateral ligament [UCL], rotator cuff, superior labrum anterior-posterior/labral, acromioclavicular joint, biceps tendinopathy, ulnar neuritis, posteromedial impingement, flexor-pronator, latissimus dorsi/teres major), and management (rehabilitation/physical therapy, arthroscopy, UCL reconstruction/repair/internal brace, return to play); pediatric/adolescent studies and nonoverhand sports were excluded. Studies were screened for inclusion by 2 reviewers and synthesized qualitatively due to heterogeneity.

**Results:**

Significant differences in timing, velocity, and arm angles were observed between baseball and football throwing motions, which affected injury prevalence and nature. Baseball pitchers demonstrated higher rates of both elbow and shoulder injuries compared to football quarterbacks, with elbow injuries being particularly prevalent due to the high volume of repetitive throwing motions. Treatment options ranged from nonoperative approaches, including physical therapy, to operative interventions such as UCL reconstruction.

**Conclusion:**

The biomechanics of throwing in baseball and football differ significantly, leading to distinct injury patterns between sports. Baseball pitchers experience more shoulder and elbow injuries due to repetitive high-velocity throwing, while quarterbacks typically sustain trauma-related injuries rather than overuse injuries.

Throwing is a complex skill that requires the coordination of several body segments to generate and transfer forces efficiently.^14^ Quantifiable and qualitative differences exist between the throwing mechanics of each sport, specifically baseball and football.^30^ In baseball, pitchers are required to throw with maximal velocity and accuracy from a consistent base,^36^ while in football, quarterbacks must continually adapt their base mechanics to throw with accuracy and timing to hit a moving receiver, occasionally having to scramble^30^ and vary their arm slots, while also evading defensive pressure.

Various factors implicated within the throwing mechanics of both baseball and football affect the mechanics of the thrower and, thus, the injuries associated with each sport; for example, due to the heavier weight of a football compared to a baseball, quarterbacks in football are unable to generate as much shoulder internal rotation velocity compared to baseball pitchers.^30^

Throwing biomechanics in baseball and football have been well studied. In baseball pitching, there are distinct throwing phases: wind-up, early cocking, late cocking, acceleration, deceleration, and follow-through.^13,18,20,21,24,26,50,61^ Quarterbacks in football have similar mechanics to baseball pitchers. The shoulder has the most degrees of freedom among all joints in the body, with both static and dynamic structures, such as the labrum, rotator cuff, and additional muscles surrounding the glenohumeral joint,^3,25,31,40,46,66^ and it is often subject to significant strain. Thus, football quarterbacks experience similar forces on the shoulder as baseball pitchers.^25^ Despite the similarities in the forces exerted on the shoulder, there are distinct throwing phases between both sports, with baseball pitching having a component of stationary momentum buildup through a windup, a longer stride, and a distinct pattern of deceleration developing through the kinetic chain that necessitates the need for rotator cuff muscle activation, as well as activation of the gluteal muscles that help both help decelerate the arm and “launch” the baseball out of a pitcher's hand.^26,30^ These varying mechanics, in turn, create stress and tension in various parts of the arm due to these underlying biomechanical differences in mechanics. Injuries in baseball are often attributed to the high velocities and extreme torque placed on various parts of the arm, resulting in injuries that are often localized in the elbow and the shoulder. Baseball injuries typically consist of ulnar collateral ligament (UCL) injuries and rotator cuff tears, among several others, and are typically localized to the elbow and shoulder.^48,60^ Injuries sustained by quarterbacks typically result from overuse due to repeated throwing and occur in various locations along the arm.^41^

While injuries resulting from high-velocity baseball throwing have been previously examined, there is a lack of studies examining injuries associated with quarterbacks’ throwing mechanics. Understanding the injuries resulting from throwing a football will illuminate the future of injuries within sports—especially given the evolving nature of elite quarterbacks throwing from varying arm slots, on the run, and from longer distances across their bodies. Further, understanding how biomechanics differ between baseball and football will help provide context for injuries stemming from throwing—highlighting distinct sites of injury—to ultimately lead to further innovation in treatment options.

This review examines the biomechanics of throwing in baseball and football—specifically in elite athletes at the semiprofessional and professional levels—and how these differences contribute to the incidence and types of upper extremity injuries commonly associated with each sport and the treatment options for various injuries.

## Biomechanical Overview of Throwing Motion

### Throwing Biomechanics in Baseball

Baseball pitching is a high-velocity, overhand throwing motion that places significant stress on the upper extremity, specifically the deltoid and the rotator cuff muscles.^11^ The throwing motion consists of 6 phases: wind-up, early cocking, late cocking, acceleration, deceleration, and follow-through.^8,30^ The arm motion in baseball pitching is characterized by rapid external rotation of the shoulder, followed by a rapid internal rotation, creating a “whip-like” motion that generates high ball velocity.^1^

Pitching kinematics and kinetics play a significant role in the biomechanics of a baseball pitch, specifically in the onset of injuries. The throwing arm's path is unique, starting behind the body in a wind-up phase and ending with the follow-through phase across the body's opposite side. The arm-cocking phase of throwing a baseball places significant stress on the shoulder, as this phase produces maximum anterior shoulder force, horizontal adduction torque, internal rotation torque, and elbow varus torque^31^; forces are highest on the deltoid muscle in early cocking and on the rotator cuff musculature in late cocking. The arm acceleration phase consists of maximum elbow flexion torque, and arm deceleration consists of maximum proximal shoulder force and proximal elbow force.^31^ Fortenbaugh et al^31^ indicate that these phases, consisting of maximal torque and high forces applied to varying parts of the arm and forearm, when performed repeatedly, can affect vulnerable tissue in these regions and put pitchers at risk for injury when transitioning through these susceptible positions during the pitching delivery.

Optimal pitching mechanics that will reduce biomechanical strain and risk of injury along the length of the throwing arm have been proposed previously.^19^ Diffendaffer et al^19^ indicated that at the moment the lead foot contacts the ground, the elbow flexion should have 90° of abduction, 20° of horizontal abduction, and 45° of external rotation to minimize biomechanical strain. They also indicated that the stride length should ideally be 85% of the pitcher's height, with the lead foot being slightly closed upon landing; additionally, the pelvis should be slightly rotated open toward home plate with the front shoulder facing the target (upper torso facing in the pitching direction). While these mechanics set foundational parameters for reducing biomechanical strain on the arm, these are dependent on underlying physiological mobility factors for different pitchers while discounting potential movement restrictions that are physiologically and inherently different between athletes. Thus, “ideal” mechanics are often subject to change, and the topic is in constant flux and cannot be reduced to a rigid mechanical structure.

Moreover, the importance of throwing from the “stretch” or “set” position—in which the pitcher's ipsilateral foot contacts the pitching rubber directly and the contralateral foot raises and strides toward the catcher—as opposed to the “windup”—in which the pitcher can take a step sideways or backward with the contralateral foot to generate more momentum toward the catcher—may have an impact on the incidence of upper extremity injuries of a pitcher; however, more research is needed to investigate the differences in the incidence of injuries between the “stretch” and “windup” mechanics.^64^

### Throwing Biomechanics in Football

While the differences between a baseball pitch and a football throw are stark, there are similarities between the two that are fundamental components of throwing and must be acknowledged. Biomechanical features of a football throw that are similar to a baseball pitch include external rotation of the shoulder at ball release, and shoulder abduction is also similar for throwing in both sports.^52^ Fleisig et al^30^ also note that quarterbacks “lead with the elbow” and have a decreased contribution from the lower body; “leading with the elbow” results in larger magnitudes of shoulder adduction and elbow flexion, while decreased contribution from the lower body manifests through a shorter stride, less forward trunk tilt, and less pelvis and upper torso angular velocity compared to baseball pitchers.^30^

In football, quarterbacks typically throw the ball with mechanics that differ from baseball pitching; namely, quarterbacks throw from a more upright position and have short strides.^19^ The throwing motion in football consists of similar phases to baseball, although some phases differ between the sports.^1,37^ Elbow flexion during throwing is typically greater for quarterbacks, and the follow-through is shorter due to the possibility of impact from an opposing player.^44^

Otherwise, the football throw consists of an arm-cocking phase, an arm acceleration phase, and a follow-through phase.^44^ In an experiment done by Fleisig et al,^30^ the authors noted that a football quarterback displayed greater elbow flexion than pitchers, with an average of 113°. The maximum medial force on the elbow during the arm-cocking phase was shown to be 280 N with a maximum varus torque of 54 N·m. To decelerate the elbow, quarterbacks generate a flexion torque of 41 N·m and a compressive force of 620 N.^30^

Fleisig et al^30^ noted that, compared to baseball, significantly less force and torque are produced to decelerate elbow extension in football quarterbacks. For example, multiple studies have previously quantified this notion, with elbow compressive forces during decelerations in baseball reported at 900 ± 100 N,^1,30^ 780 N,^63,64^ and 830 ± 80 N,^8,27^ whereas in football, elbow compressive forces during deceleration have been noted to be 620 ± 110 N^2,26^ and 350 ± 100 N.^24^ These studies further show that both force and torque exerted on the shoulder and elbow are lower in football than baseball across multiple phases of the throwing motion.^1,8,27,28,52,63,64^ These studies affirm that multiple factors play a role in the biomechanical mechanisms leading to throwing injuries in both sports. Furthermore, the differing surface angles (sloped vs flat) can potentially affect upstream biomechanics; however, more research is needed to examine this effect further. A comparison of the key biomechanical parameters distinguishing baseball pitchers from football quarterbacks is summarized in Table 1.

**Table 1:** Comparison of Throwing Biomechanics: Baseball Pitchers Versus Football Quarterbacks

Characteristic	Baseball Pitcher	Football Quarterback
Ball weight	~5 oz (142 g)	~14-15 oz (397-425 g)
Throwing surface	Sloped mound	Flat ground
Arm angle	Consistent, trained overhead or three-quarter slot	Variable; often overhead, adjusted by situation
Stride length	Long (approximately 85% of height)	Shorter stride
Shoulder external rotation	Very high (can reach 170°-180° in late cocking phase)	Moderate to high (~150°)
Peak elbow torque	High (~120 N·m during late cocking phase)	Lower (~54 N·m)
Lower body involvement	High (pelvic rotation, glute and core activation drive momentum)	Less consistent; varies with movement and pocket collapse
Torso angular velocity	High (greater trunk rotation and tilt toward target)	Lower trunk rotation and forward tilt
Arm-cocking elbow flexion	90°-100° elbow flexion	Greater average elbow flexion (~113°)
Follow-through	Full and extended; essential for deceleration and injury prevention	Often truncated due to risk of contact
Common throwing phases	Six phases: wind-up, early cocking, late cocking, acceleration, deceleration, follow-through	Typically 3 defined phases: cocking, acceleration, follow-through
Injury risk regions	Elbow (ulnar collateral ligament), shoulder (rotator cuff, labrum), due to high velocity and torque	Shoulder (acromioclavicular joint, rotator cuff), biceps tendon; often traumatic, less overuse

## Upper Extremity Injuries Resulting From Throwing

### Upper Extremity Injuries in Baseball

Upper extremity injuries are the most common type of injury in Major League Baseball, comprising 51.4% of all injuries.^51^ Shoulder and elbow injuries in baseball pitchers are common due to the high, repetitive forces placed on the shoulder and elbow during the throwing motion. Injuries most commonly include rotator cuff tears,^48^ labral tears,^49^ UCL injuries, and medial epicondylitis.^60^ A meta-analysis across PubMed, MEDLINE, and Embase performed by Tramer et al^59^ found the greatest upper extremity injury incidence rates to be hand/wrist (150.3 per year), forearm flexor strains (26.8 per year), primary UCL injury (0.23-26.8 per year), acromioclavicular (AC) joint injuries (18.4 per year), latissimus dorsi and teres major injuries (7 per year), labral tears (1.3-7 per year), and shoulder dislocations (0.05 per year).

Peak shoulder external rotation during the late cocking phase is a key movement in the pitching motion that contributes to shoulder injury risk.^31,43^ Rotator cuff tears can be caused by acute tensile overload or repetitive trauma, the latter of which contributes to a cascade of adaptive changes.^43^ Labral tears are implicated in the late cocking phase of the throwing motion; in this phase, increased posteriorly directed strain and twisting of the biceps at maximum external rotation can lead to a superior labrum anterior-posterior (SLAP) tear.^43^ Type II SLAP tears, consisting of labral fraying with a detached biceps tendon anchor, are the most common labral pathology and are associated with repeated overhead throws.^35,56,57^

Elbow injuries in baseball are often caused by repetitive throwing as a part of pitching.^28^ It has been well established that the kinetic chain implicated in baseball pitching generates significant forces to produce high pitching velocities, especially in professional pitchers.^4^ The high levels of energy generated throughout the kinetic chain in the delivery of a pitch and the tremendous forces that are exerted on the arm have typically been considered the cause of repetitive throwing injuries, especially at the elbow.^4^ Specifically, the elbow is at the highest risk for injury during the late cocking phase; in this phase, maximum external valgus of the elbow and maximum external shoulder rotation occur, producing significant torque of up to 120 N·m at the elbow.^4,27,29,61,62^ Significant torque generated at the elbow is often correlated with injuries, such as tears of the UCL.^63^ The magnitude of this torque, referred to as elbow-varus torque, is larger and more strongly associated with higher pitching velocities within an individual pitcher.^55^ The study performed by Slowik et al^55^ found, however, that the correlation between higher elbow-varus torque and high pitching velocity is not effectively comparable across various pitchers due to confounding effects of pitcher-specific differences.

UCL tears, in particular, have become increasingly more prevalent, especially in younger-aged minor league baseball players.^9^ This is a common trend seen at the professional level with elite baseball pitchers, all the way up to the Major League level; this is an especially important point to note, as the widely disseminated notion is that UCL injuries are on the decline, which has been refuted.^9,17^ The injury itself may be attributed to the increased focus on high pitching velocities and maximizing biomechanics to achieve maximum force generation and, thus, increased pitching velocities. Specifically, the tears are attributed to overuse and stress on the UCL due to extreme valgus stress during the late cocking and acceleration phase.^2,4,29,33,63^ Furthermore, Chalmers et al^10^ found that pitching velocity was the strongest predictor of UCL tears and, hence, UCL reconstruction. The authors also found that subsequent UCL reconstructions were needed for pitchers with higher average pitching velocities; they also indicate that these pitchers may need to focus on mechanics to reduce the risk of UCL tears. However, future work should look into the extent to which highly optimized biomechanics may actually be contributing to injury by creating a positive feedback loop by increasing pitching velocity, thus contributing to UCL tears by the mechanism described by Chalmers et al.^10^

Additional injuries occur in the elbow and surrounding regions as well and can be attributed to the extreme forces and torques on the shoulder and elbow joint during the pitching delivery. Additional elbow injuries stemming from throwing a baseball include tendon injuries (eg, biceps and triceps tendons), ulnar nerve injuries, posteromedial impingement, and bone fracture.^15^ The repetitive throwing motion contributing to elbow injuries can be compounded by poor throwing biomechanics, which exacerbates the torque and force produced on the elbow during each pitch.^28^

### Upper Extremity Injuries in Football

Common injuries seen in quarterbacks are rotator cuff tears, biceps tendinitis, and labral tears.^41^ However, rather than injuries stemming from repetitive trauma, injuries to National Football League (NFL) quarterbacks often are acute in nature, such as from traumatic contact.^22^ The mechanism of injury in football throwing is different from baseball throwing, as quarterbacks typically throw from a more overhead arm slot.^38^ The throwing motion in football places less stress on the shoulder and elbow than baseball throwing, but it still requires significant coordination and timing between the upper extremity segments.^54^ Kelly et al^38^ used the NFL Injury Surveillance System between 1980 and 2001 to demonstrate that only 14% of all quarterback injuries were attributed to overuse, and the most common was rotator cuff tendinitis (6.1%), followed by biceps tendinitis (3.5%).

Furthermore, studies show that NFL quarterbacks demonstrate elevated rates of shoulder injuries compared to other position players, with 71% of quarterbacks reporting a history of shoulder injury compared to 50% of all NFL athletes.^5^ Injuries to the shoulder girdle can be related to trauma or the quarterback's throwing motion. Kelly et al^38^ found that 82.3% of NFL quarterback shoulder injuries were related to direct contact (AC joint sprain and dislocation, shoulder dislocation and instability, SLAP and labral tears, contusions and fractures), and 14% of shoulder injuries occurred secondary to throwing (rotator cuff tendinitis and biceps tendinitis).^41^ AC joint injuries were the most common type of shoulder injury, representing 40% of all cases tracked by the NFL Injury Surveillance System.^38^ Elbow injuries—and, in particular, injuries to the UCL—have also been observed, although most occur from traumatic contact as opposed to the throwing motion itself,^22^ with very few football quarterbacks having to undergo UCL surgery, although it has been noted previously.^58^

Limited examinations of the mechanism of throwing injuries that result from overuse in quarterbacks have been performed. Thus, more research is necessary to examine throwing injuries stemming from throwing mechanics and upper extremity overuse, especially given the dynamic nature of throwing mechanics in football.

## Treatment for Upper Extremity Injuries

### Nonoperative Treatment

Nonoperative treatment has been an integral part of treating throwing injuries in various sports. Many times, the treatment for such injuries, such as UCL tears, entails nonoperative and minimally invasive approaches for nonthrowing athletes and low-demand patients.^42^ Such treatment has been successful in treating nonthrowing NFL players.^39^

Physical therapy is the first-line treatment for most upper extremity throwing injuries; the goal, initially, is to reduce pain and inflammation while attempting to stabilize and recorrect range of motion, muscle balance, and joint stability.^65^ Physical therapy is usually followed by various phases of strengthening exercises to enable the athlete to return to prior form.^65^ Wilk and Arrigo^65^ discuss return-to-throwing programs for baseball pitchers following physical therapy and strengthening, given that ample time has passed for the injury to heal. Similar nonoperative treatment is ideal for all throwing athletes, given that the injury sustained is not severe enough to necessitate surgery. Dodson et al^22^ found that among 10 studied cases of UCL injuries among quarterbacks over a 14-year period, 9 were treated nonoperatively, leading the authors to suggest that professional quarterbacks sustaining UCL injuries may be able to return to competition through nonoperative treatment and recovery unless the injury involves a complete tear.

Further, nonoperative treatment for nagging injuries has been a commonly used methodology in the past; in 2008, it was found that 93% of NFL physicians used ketorolac injections prior to a game to alleviate pain and discomfort from injuries.^53^ However, ketorolac injection utilization has since declined, as only 48% of NFL physicians administered ketorolac in 2016, which was likely due to more effective pain management strategies. This suggests that physicians may be looking to treat the root of the problem through operative methods as opposed to alleviating pain in elite athletes, which may be related to the intensive demands of an elite, high-level athlete to achieve peak performance at all times.^53^

NFL players who underwent surgical treatment for AC joint injuries faced longer times to return to play than players treated with nonoperative injury management.^45^ When surveyed, most NFL team physicians preferred to treat type III AC joint injuries nonoperatively (72%), reserving surgical intervention (28%) only for injuries to a quarterback's throwing arm.^53^

However, for elite overhead throwing athletes, such as baseball pitchers and football quarterbacks, nonoperative treatment has been observed to be an unsuccessful mode of treatment to enable a throwing athlete to return to prior form; in such instances, operative treatment may be necessary.^39^

### Operative Treatment

Injuries that occur from throwing can be treated either nonoperatively or operatively, depending on the type of injury and the desired outcomes of a patient. However, if an injury is severe, surgery may be the ideal route to enable elite athletes to return to their baseline athletic performance.

Despite the success of nonoperative treatment for throwing injuries, injuries to the shoulder or elbow in throwing athletes requiring surgery are relatively common.^12^ The most common sites for injury for a throwing athlete include the shoulder and the elbow. As discussed previously, these injuries include SLAP tears, rotator cuff injuries, and labral tears in the shoulder, as well as UCL tears, epicondylitis, and loose body formation in the elbow.^16^ Surgery is typically necessary if an athlete does not successfully respond to nonoperative treatment, and the goal is to return to the preinjury level.^16^

Cohen et al^16^ note that during a 4-season period of professional baseball in a single club, 19 shoulder procedures were performed on pitchers and 8 procedures on position players, with most procedures involving labral tears (23 athletes). Most of these injuries were a result of microtrauma involved in the repetitive throwing motion and consisted of type II SLAP lesions that failed a trial of initial nonoperative management. The remainder of procedures addressed full- or partial-thickness rotator cuff tears, impingement, or capsular contracture. The surgical treatments for these injuries consisted of suture anchor repair and labral debridement for type II SLAP tears; arthroscopic repair and debridement for full- and partial-thickness rotator cuff tears, respectively; subacromial decompression for impingement symptoms; and capsular release for contracture. All procedures performed in the study were conducted arthroscopically by surgeons with fellowship training in sports medicine. Among the 27 athletes with operative shoulder injuries, 16 returned to professional baseball, and 11 retired as a result of their injuries.^16^

One of the most common injuries requiring surgery in throwing athletes, especially baseball pitchers, occurs at the UCL in the elbow.^7^ The surgery, which involves reconstruction of the UCL, is commonly known as “Tommy John surgery.”^47^ Although UCL reconstruction—first pioneered by surgeon Frank Jobe—was initially considered an experimental treatment option, it has now not only turned into the standard of care for UCL tears to return to prior athletic form^58^ but may also lead to improved performance.^24^ The operation itself has evolved tremendously since it was first performed in 1974 by Frank Jobe, and it is now a multifaceted procedure that uses multiple techniques to improve biomechanical strength and accelerate the rate of return to elite athletic form and competition.^32^ The most common technique in UCL reconstruction is known as the docking technique, and iterations of the technique have been explored, such as combining the docking technique with a proximal single-tunnel suspensory fixation technique.^32^

In addition to UCL reconstruction, Cohen et al^16^ explored additional surgical elbow procedures performed on athletes, including arthroscopic and open loose body excision, debridement of flexor-pronator mass tears, and submuscular ulnar nerve transposition for athletes with ulnar neuritis. Although UCL reconstruction has been the gold standard for rehabilitating UCL tears, the lengthy recovery timeline, with an average 11.6-month recovery for professional baseball athletes, poses challenges in returning to play.^6^ Innovations in UCL repair surgery, such as internal bracing, have the potential to provide less invasive alternatives to UCL reconstruction and expedited recovery timelines due to improved stability.^34^ In a study of 350 athletes who underwent UCL internal bracing, Wilk et al^67^ found an average return-to-play recovery period of 7 months. These findings were further corroborated by Dugas et al,^23^ who found an average return to play of 6.7 months following UCL repair using an internal brace.

A summary comparison of common throwing-related upper extremity injuries, their treatment modalities, and return-to-play timelines in baseball and football athletes is provided in Table 2.

**Table 2:** Comparison of Treatment Approaches and Return-to-Play Outcomes for Common Throwing Injuries in Baseball and Football*^*a*^*

Injury Type	Athletes Affected	Mechanism of Injury	Nonoperative Treatment	Operative Procedure(s)	Return-to-Play Time	Return to Prior Level (%)	Notable Complications
UCL tear	Baseball (pitchers); football (QB, uncommon)	Repetitive valgus stress in late cocking and acceleration (≈120 N·m), overuse; traumatic contact far less common in QBs	First-line rest, PT, progressive throwing; 9/10 NFL QBs in 1 series succeeded nonoperatively	UCL reconstruction (Tommy John, docking variants) ± proximal suspensory fixation; UCL repair with internal brace	~11.6 months (reconstruction); 6.7-7 months (internal brace repair)	High; most athletes regain preinjury level (multiple studies report successful return)	Ulnar neuritis, graft failure, need for revision
AC joint injury	Baseball (infrequent); football (QB, common)	Overuse rare; direct shoulder contact—82% of QB shoulder injuries	Preferred for type III injuries in 72% of teams; immobilization, PT, analgesia	Surgical AC joint reconstruction reserved for throwing arm/type III+ lesions	Surgical cases return slower than nonoperative (time not specified)	—	Hardware irritation; prolonged pain or instability if surgery delayed
SLAP tear	Baseball (pitchers); football (QB due to contact)	Peel-back and torsional strain on biceps anchor at maximum ER in late cocking; contact trauma in QBs	Initial PT and posterior capsular stretching; many elite throwers progress to surgery after failed rehab	Arthroscopic suture anchor repair or labral debridement	For football, overall 16/27 (59%) returned to pro play after labral-dominant shoulder surgery	≈59% in study (football)	Shoulder stiffness, persistent pain leading to retirement (11/27 in same series)
Rotator cuff tear	Baseball (pitchers); football (QB due to overuse or trauma)	Acute tensile overload or cumulative micro-trauma during cocking and deceleration phases	Structured PT focusing on cuff strength and scapular control (first-line for most shoulder injuries)	Arthroscopic repair (full/partial) ± subacromial decompression	—	About half of surgically treated shoulders (mixed labrum/cuff) returned (16/27)	Retear, residual weakness, impingement
Biceps tendinitis	Baseball (pitchers); football (QB)	Repetitive overhead throwing leading to long head of the biceps inflammation (overuse)	Conservative care: rest, NSAIDs, PT (general first-line strategy)	—	—	—	Possible progression to SLAP lesion if unaddressed

a AC, acromioclavicular joint; ER, external rotation; NFL, National Football League; NSAIDs, nonsteroidal anti-inflammatory drugs; PT, physical therapy; QB, quarterback; SLAP, superior labrum anterior-posterior; UCL, ulnar collateral ligament.

## Concluding Remarks and Future Outlook for Research

The biomechanics of throwing in baseball and football differ significantly, leading to distinct injury patterns between sports. Baseball pitchers experience more shoulder and elbow injuries due to repetitive high-velocity throwing, while quarterbacks typically sustain trauma-related injuries rather than overuse injuries.
